# Analysis of the Potential Role of GluA4 Carboxyl-Terminus in PDZ Interactions

**DOI:** 10.1371/journal.pone.0008715

**Published:** 2010-01-14

**Authors:** Sarah K. Coleman, Chunlin Cai, Nisse Kalkkinen, Esa R. Korpi, Kari Keinänen

**Affiliations:** 1 Department of Biosciences, Division of Biochemistry, Viikki Biocenter, University of Helsinki, Helsinki, Finland; 2 Institute of Biotechnology, Viikki Biocenter, University of Helsinki, Helsinki, Finland; 3 Institute of Biomedicine, Pharmacology, University of Helsinki, Helsinki, Finland; Medical College of Georgia, United States of America

## Abstract

**Background:**

Specific delivery to synapses of α-amino-3-hydroxy-5-methylisoxazole-4-propionate (AMPA) receptors with long-tailed subunits is believed to be a key event in many forms of activity-dependent changes in synaptic strength. GluA1, the best characterized long-tailed AMPA receptor subunit, contains a C-terminal class I PDZ binding motif, which mediates its interaction with scaffold and trafficking proteins, including synapse-associated protein 97 (SAP97). In GluA4, another long-tailed subunit implicated in synaptic plasticity, the PDZ motif is blocked by a single proline residue. This feature is highly conserved in vertebrates, whereas the closest invertebrate homologs of GluA4 have a canonical class I PDZ binding motif. In this work, we have examined the role of GluA4 in PDZ interactions.

**Methodology/Principal Findings:**

Deletion of the carboxy-terminal proline residue of recombinant GluA4 conferred avid binding to SAP97 in cultured cells as shown by coimmunoprecipitation, whereas wild-type GluA4 did not associate with SAP97. Native GluA4 and SAP97 coimmunoprecipitated from mouse brain independently of the GluA1 subunit, supporting the possibility of *in vivo* PDZ interaction. To obtain evidence for or against the exposure of the PDZ motif by carboxyterminal processing of native GluA4 receptors, we generated an antibody reagent specific for proline-deleted GluA4 C-terminus. Immunoprecipitation and mass spectrometric analyses indicated that the carboxyl-terminus of native GluA4 AMPA receptors is intact and that the postulated single-residue cleavage does not occur to any significant extent.

**Conclusion/Significance:**

We conclude that native GluA4 receptors are not capable of canonical PDZ interactions and that their association with SAP97 is likely to be indirect.

## Introduction

AMPA receptors mediate the majority of mammalian fast excitatory neurotransmission and play important roles in synaptic plasticity. AMPA receptors are tetramers composed of various combinations of four homologous subunits GluA1 to 4 (alternatively GluR-A to -D, or GluR1-4). The number and subunit composition are important determinants of synaptic strength. Subunit-specific AMPA receptor trafficking is largely governed by the cytoplasmic C-terminal tails (CTDs) (reviewed in [1], [2]); with the specific activity-dependent insertion of the long-tailed (70–80 residues) subunits GluA1, GluA2_L_ (minor isoform) and GluA4 occuring in various forms of synaptic plasticity, leading to enhanced AMPA receptor responses [3]–[7].

Due to its central role in hippocampal CA3-to-CA1 pyramidal cell synapses [8], [9] GluA1 trafficking has received more attention than either GluA4 and GluA2_L_. The C-terminus of GluA1 has a canonical class I PDZ motif and mutations at this motif prevent activity-dependent transport of GluA1 to synapses in hippocampal slice preparations and in cultured neurons [4], [7], [10]. Interestingly, the total elimination of the PDZ motif, by a seven-residue deletion, leaves GluA1-dependent synaptic plasticity intact [11], [12], suggestive of a complex interplay between multiple C-terminal PDZ- and non-PDZ interactions. Reported PDZ interaction partners for GluA1 include SAP97 [13]–[16], mLin-10 [17], syntenin [18] and Shank3 [19]. Notably, overexpression of SAP97 drives GluA1 to synapses and occludes long-term potentiation [16], [20], whereas its RNAi knockdown reduces surface levels of GluA1 in neurons [20]. Moreover, SAP97 is present in the postsynaptic membrane and has a peripheral localization in the postsynaptic density, consistent with a role in the insertion or stabilization of AMPA receptors [21]–[23].

While less studied than GluA1, both GluA4 and GluA2_L_ are also implicated in synaptic plasticity [5], [6], [24]. Direct protein interactions of GluA4 CTD include 4.1N [25], PKCγ [26], α-actinin-1 and IQGAP1 [27], but none of these involve the extreme carboxy terminus. The CTDs of GluA4 and GluA2_L_ and share several sequence characteristics with GluA1, including the residues corresponding to the PDZ motif. However, one extra residue, proline in GluA4, and serine in GluA2_L_, blocks the PDZ motif. Given the existence of cytosolic carboxypeptidases in the nervous system [28], the possibility thus arises that limited proteolytic cleavage of GluA4 C-terminus could expose the PDZ motif *in vivo* to faciliate interactions with PDZ domain-containing trafficking proteins. An analogous mechanism involving enzymatic removal of a single C-terminal residue operates in the detyrosination of α-tubulin in the brain and peripheral tissues [28], [29]. AMPA receptor cytoplasmic tails are subject to proteolytic cleavage in hippocampal slices following stimulation [30], and both calpain [31]–[33] and caspase-8 [34] mediated cleavage of GluA1 C-terminal tail have been suggested to play a role in excitotoxic processes following raised intracellular Ca^2+^ levels.

In the present study, we analyzed the interaction of GluA4 with SAP97 in cultured cells and *in vivo*, specifically addressing the hypothesis that the carboxy-terminus of native GluA4 is subject to proteolytic processing to reveal the PDZ motif. We show that native GluA4 is associated with SAP97, independently of its coassembly with the GluA1 subunit. However, immunochemical and mass spectrometric analyses indicate that the hypothesized cleavage of GluA4 C-terminus by endogenous enzymatic activity in neurons does not occur to any detectable extent, and therefore, the association with SAP97 is likely to be indirect and/or mediated by non-PDZ mechanisms.

## Results and Discussion

**Figure 1:**
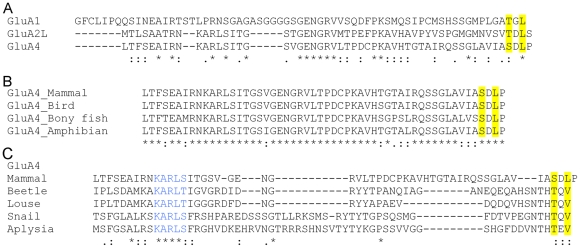
Sequence characteristics of long-tailed AMPA receptor subunits. (A) Alignment of the unique carboxyterminal extensions of rat long-tailed GluA1, GluA2_L_ and GluA4 subunits. The accession codes (SwissProt/TrEMBL) for the sequences are: GluA1, P19490; GluA4, P19493; GluA2_L_, P23819-3. (B) Conservation of GluA4 C-terminal sequence in vertebrate evolution. The indicated GluA4 orthologs represent diverse vertebrate lineages: mammals (*Rattus norvegicus*, rat, P19490), birds (*Gallus gallus*, chicken, Q90858), bony fishes (*Danio rerio*, zebra fish, Q71E58) and amphibians (*Xenopus tropicalis*, western clawed frog; the sequence represents a virtual translation of Genbank EST CX366243). (C) Alignment of mammalian GluA4 carboxyterminal sequence with its closest invertebrate homologs: Mammal (rat); Beetle, (*Tribolium castaneum*, Red flour beetle, XP 968786); Louse, (*Pediculus humanus corporis*, human body louse, XP 002430327); Snail (*Lymnaea stagnalis*, great pond snail, CAA42683); Aplysia (*Aplysia californica*, California sea har, ABB03888). In all alignments, the residues conforming to the class I PDZ motif (-Thr/Ser-X-Φ; Φ denoting an amino acid residue with large aliphatic side chain, X standing for any amino acid) are highlighted in yellow. Asterisks indicate identical residues, whereas strong and weak similarities (according to Gonnet Pam250 matrix [49]) are indicated by colons and dots, respectively.

The sequence similarities in the CTDs of the long-tailed AMPA receptor subunits are illustrated in Figure 1. In rat GluA4 and GluA2_L_, the residues corresponding to the class I PDZ motif in GluA1 are conserved, but followed by a single “blocking” residue (Figure 1A). The extreme carboxyterminal tetrapeptide sequence, including the cryptic PDZ motif and the blocking residue, are absolutely conserved in GluA4 orthologs representing widely disparate vertebrate lineages (Figure 1B). This is consistent with important function(s), most likely, C-terminus -dependent protein interactions, but none such are known. The carboxyl-terminal tail of GluA2_L_ shows a similar high degree of sequence conservation (Figure S1). Interestingly, the invertebrate homologs showing the closest similarity to GluA4 C-terminus have a genuine class I PDZ motif with no blocking residue (Figure 1C). Importantly, the sequence pattern KARLS/T, a distinguishing feature of GluA2_L_ and A4 subunits as opposed to GluA1, is present in the invertebrate sequences, arguing for a closer kinship to GluA4/2_L_. These findings raise the possibility that the blocking residue in vertebrate “non-GluA1” long-tailed AMPA receptor subunits may have evolved to faciliate its regulated exposure to PDZ interactions.

**Figure 2:**
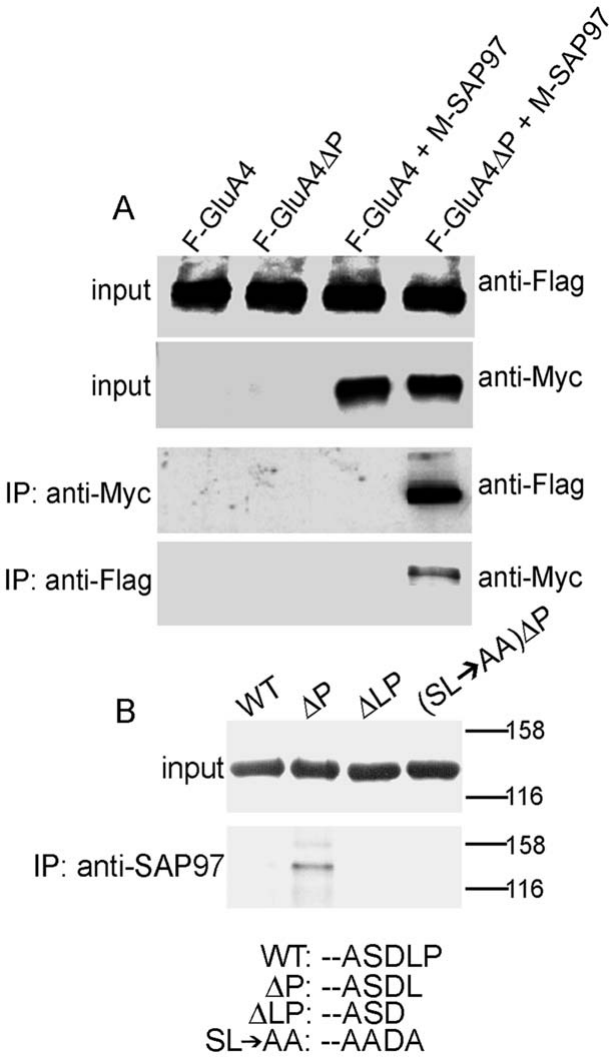
Deletion of proline-902 exposes a functional PDZ motif in GluA4 and confers binding to SAP97. (A) Expression of wild-type or mutant GluA4, with or without co-expressed myc-tagged SAP97 in HEK293 cells. Upper panels show expression of all proteins; lower panels show co-immunoprecipitation of GluA4ΔP, but not full-length GluA4 with SAP97. Immunoblotting antibodies are indicated on right. (B) Transiently expressed GluA4ΔP can co- immunoprecipitate with endogenous SAP97 from HEK293 cells. Upper panel shows similar expression levels of transfected GFP-tagged constructs. Lower panel show immunoprecipitation with anti-SAP97 specific antibody. Both blots were probed with anti-GFP IgG. The extreme carboxyterminal sequences of the expressed proteins are shown below.

A previous study using bacterially expressed protein domains demonstrated that removal of the extreme C-terminal proline resulted in avid binding of GluA4 CTD to PDZ domains of SAP97 under *in vitro* conditions with purified proteins [13]. To investigate the potential functionality of GluA4 cryptic PDZ motif in living mammalian cells, full-length wild-type (wt) and mutated flag-tagged GluA4 were expressed in transfected HEK293 cells together with myc-tagged SAP97. Coimmunoprecipitation from detergent extracts of cell homogenates showed no association between wt GluA4 and SAP97, whereas the GluA4 mutant lacking proline-902 (GluA4ΔP) immunoprecipitated as a complex with SAP97 (Figure 2A). Next, we wished to determine if GluA4ΔP also associated with the endogenously expressed SAP97 in HEK293 cells, and whether the interaction shows features typical of class I PDZ interactions. Thus, HEK293 cells were transfected for expression of N-terminally GFP-tagged GluA4 constructs, followed by immunoprecipitation with anti-SAP97 sera. Consistent with the above results GFP-tagged GluA4ΔP, but not the wild-type receptor, co-precipitated with SAP97. Disruption of the exposed PDZ interaction motif, either by further deletion of the carboxyl-terminal Leu-901 (GluA4ΔLP) or by mutation of Ser-899 and Leu-901 to alanine [GluA4(SL→AA)ΔP], abolished the interaction (Figure 2B). This is in agreement with the requirement for a large aliphatic side chain at the carboxy-terminal position for class I PDZ interactions.

**Figure 3:**
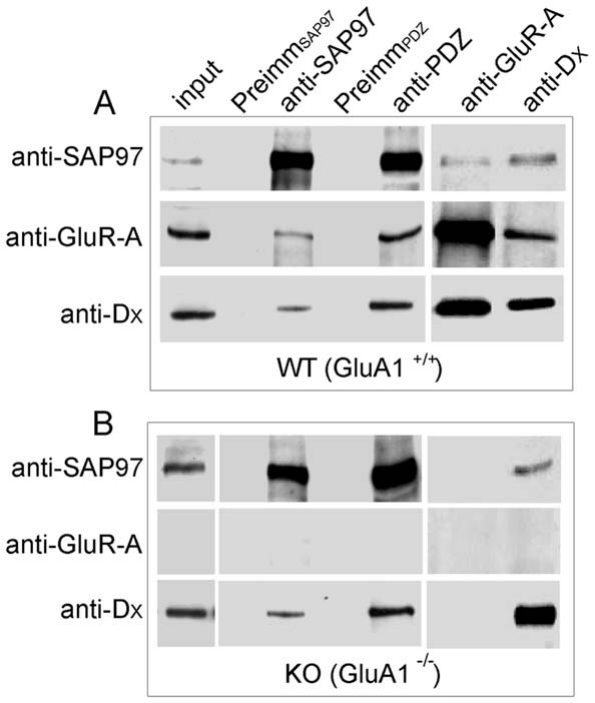
Native GluA4 AMPA receptors interact with SAP97. Whole brain detergent extracts prepared from (A) wild-type (WT, GluA1^+/+^) and (B) GluA1 knockout mice (GluA1^−/−^) were subjected to immunoprecipitation. Immunoprecipitating antibodies are indicated on top; whereas antibodies used for detection of the immunocomplexes are shown on the left.

Having confirmed the potential capability of proline-deleted GluA4 for PDZ interactions with SAP97, we next wished to determine whether native GluA4 receptors associate with SAP97 or with related PDZ proteins. Extracts from whole brain homogenates of adult mice were immunoprecipitated with anti-SAP97 or anti-PDZ, a broadly specific antiserum recognizing all four postsynaptic density-95 (PSD-95) family membrane-associated guanylate kinase homologs (Maguks) (Figure S2). The corresponding preimmune sera served as specificity controls. Immunoblot analysis, performed with antibody specific for GluA4 N-terminal domain (anti-D_x_ [25]) showed the presence of GluA4 in both anti-SAP97 and anti-PDZ immunoprecipitates (Figure 3A). GluA1, employed as a positive control, coimmunoprecipitated with SAP97 as expected. Substantial coimmunoprecipitation of GluA4 and GluA1 was also noticed, consistent with the existence of GluA1/4 heteromeric receptors (Figure 3A). In principle, coassembly with GluA1 subunit would lead to physical association of GluA4 subunits with SAP97 without any direct interaction [14]. To determine if this is the case, immunoprecipitations were also performed from mutant mice lacking GluA1 expression (GluA1^−/−^; [9]). Immunoreactive GluA4 was prominently present in anti-SAP97 and anti-PDZ immunoprecipitates prepared from brains of GluA1^−/−^ mice, indicating that GluA4 can associate with SAP97 independently of GluA1 (Figure 3B). Conversely, SAP97 was present in the GluA4 immunoprecipitates prepared from both wild-type and GluA1 knockout mice (Figure 3A,B).

If the *in vivo* association of GluA4 with SAP97 was due to the exposure of its cryptic PDZ motif by a cellular carboxypeptidase(s), GluA4 preparations isolated from native tissue should contain modified C-termini with potentially identifiable protein chemical or immunochemical signatures. To address this possibility, we utilized MALDI-TOF mass spectrometry to analyze tryptic digests of the ∼100-kDa receptor band obtained from GluA4 immunoprecipitates prepared from rat brain homogenates. Two different antibodies were employed, anti-BD_L_, a rabbit polyclonal antibody recognizing the long CTDs of both GluA4 and GluA2 subunits [35] and Fab7, a monoclonal antibody specific for a conformational epitope present in the N-terminal domains of GluA4 and GluA2 subunits [36]. However, mass spectrometric analysis failed to identify any C-termini of either wild-type or modified versions of GluA4 in spite of an abundance of peptides corresponding to other regions of the receptor polypeptides (Table 1 and Table S1).

**Table 1:** MALDI-TOF mass spectrometric peptide fingerprint analysis of the 100-kDa protein band present in the immunoprecipitates.

Immunoprecipitating antibody	Primary match	Intensity coverage
Anti-BD_LONG_ IgG	AMPA receptor subunits	71.4%
Fab 7	AMPA receptor subunits	77.9%
Anti-DΔP IgG	Dynamin 1	79.2%

**Figure 4:**
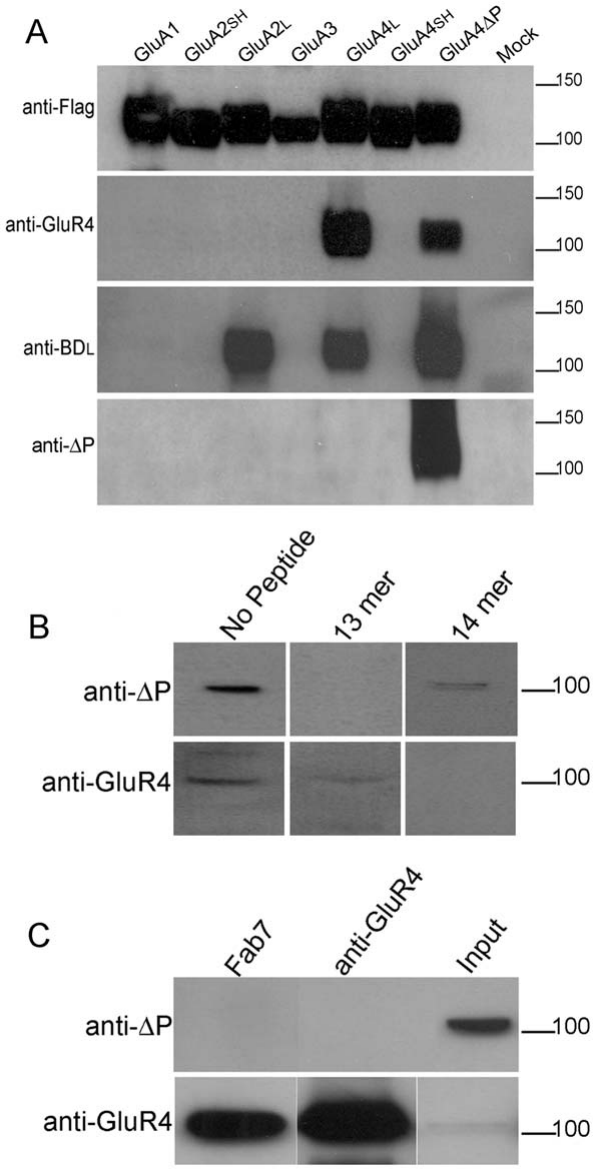
Analysis of AMPA receptors with an antibody specific for the exposed PDZ motif in GluA4ΔP. (A) HEK293 cells expressing flag-tagged AMPA receptor subunits with all potential wild-type CTDs and the mutant GluA4ΔP (indicated above) were immunoblotted with the antibodies indicated to the left. Short-tailed isoforms of GluA2 and GluA4 are indicated by SH. The initial antiserum, anti-BD_L_ detects both A2 and A4 long tails isoforms and GluA4ΔP. After the depletion procedure and purification, the anti-ΔP IgG only recognises GluA4ΔP (lower panel). (B) Anti-ΔP IgG labels a single 100 kD band in rat cerebellar tissue; this is specifically blocked by preincubation with 13mer peptide (upper panels). Similarly anti-GluR4 IgG also labels a 100 kDa band. This labelling is blocked by pre-incubation with 14 mer peptide (lower panels). (C) Immunoprecipitation from rat cerebellar extract using independent AMPA receptor antibodies fails to bring down anti-DΔP immunoreactivity (upper panel). An alternative antibody shows GluA4 levels were highly enriched in the immunoprecipitates (lower panel).

Due to the failure of direct mass spectrometric identification of GluA4 C-terminus, we decided upon an immunochemical approach. The polyclonal anti-BD_L_ antiserum was originally generated by using GluA4 CTD lacking the C-terminal proline as the antigen, and therefore it may contain also antibodies binding specifically to the extreme, processed C-terminus. However, in immunoblots, anti-BD_L_ serum recognizes GluA2_L_, GluA4 and GluA4ΔP in an equally robust manner (Figure 4A, middle panel), suggesting that antibodies specific for GluA4ΔP are a small minority. To enrich GluA4ΔP-specific antibodies potentially present in anti-BD_L_ preparations, the antiserum was repeatedly adsorbed to purified glutathione S-transferase (GST) fusion protein of wild-type GluA4 CTD, thereby depleting antibodies which bind to epitopes shared by GluA4ΔP CTD and wild-type CTD. The specificity of the final immunoreagent was determined by immunoblotting against the full range of AMPA receptor subunit CTDs. As expected, the depleted antiserum, ‘anti-ΔP’, did not any more recognize the wild-type GluA2_L_ or GluA4 subunits (or any other wild-type subunit), but reacted strongly with GluA4ΔP (Figure 4A, bottom panel). Similar specificity was observed in the immunofluorescence analysis of GluA4 and GluA4ΔP receptors expressed in transiently transfected HEK293 cells: the original anti-BD_L_ stained both GluA4 and GluA4ΔP transfectants, whilst anti-ΔP stained only the proline-deleted receptors (Figure S3A). In peptide competition experiments, binding of anti-ΔP to GluA4ΔP was abolished by a 13mer peptide corresponding to GluA4ΔP carboxyl-terminus, but not by a corresponding 14mer “wild-type” peptide (Figure S3B).

Having established an acceptable specificity for anti-ΔP antibody preparation, we used it in direct immunoblots of rat brain extracts. Anti-ΔP recognized a ∼100-kDa band, consistent with the size of GluA4 and other AMPA receptor subunits (Figure 4B). Detection of this band was fully blocked by the 13mer peptide, whereas the wild-type 14mer peptide had a much weaker inhibitory effect (Figure 4B, upper panel). Commerical GluA4-specific antibody (anti-GluR4) also recognized a band of approximately 100 kD, and in this case, the detection was weakly inhibited by the 13mer peptide and eliminated completely by the 14mer peptide (Figure 4B, lower panel). These findings are consistent with the potential existence in the brain of a subpopulation of GluA4 receptors with processed C-termini. However, direct tissue blots contain highly complex protein mixtures with the caveat that the 100-kb band may be caused by some rare crossreacting species. Therefore, we performed immunoblot analyses from preparations enriched for GluA4-containing AMPA receptors by using anti-GluR4 or Fab7 [36]. Both immunoprecipitaties reacted strongly with anti-GluR4, consistent with enrichment of native GluA4 receptors, but no anti-ΔP immunoreactivity was observed (Figure 4C). As Fab 7 recognizes an epitope in the N-terminal domain of GluA4, it should not be affected by possible C-terminal processing or protein interactions which may interfere with the binding of antibodies to carboxylterminal epitopes (like anti-GluR4). Therefore, the results suggest that native GluA4 receptors do not undergo the postulated carboxylterminal single-residue cleavage.

Finally, mass spectrometric peptide fingerprinting analysis of anti-ΔP immunoprecipitate was performed to substantiate the above results and to determine the identity of the 100-kDa anti-ΔP immunoreactive band. In contrast to the similar analysis of the anti-BD_L_ immunoprecipitated 100-kDa species (Tables 1 and S1), no AMPA receptor -derived peptides were identified. Instead, the most prominent peptides represented dynamin 1, which has a predicted size of 97.2–97.4 kDa, depending on the splice form (Tables 1 and S2). Moreover, a commercial dynamin antibody strongly labelled anti-ΔP immunoprecipitates (but not AMPA receptor immunoprecipitates) and recognised a 100 kDa band in rat cerebellar tissue (Figure S4A). Strikingly, the extreme C-terminal peptide of dynamin 1, which was also identified in the fingerprint, shares the C-terminal sequence, DL (Asp-Leu), with GluA4ΔP (Table S2). This finding suggests that the carboxyterminal DL dipeptide is the major epitope recognized by the ΔP-antibody. Consistent with this, anti-ΔP avidly binds to a fusion protein containing the carboxyterminal peptide of dynamin 1 (Figure S4B). In addition, deletion of the carboxyterminal serine from GluA2_L_, generates DL sequence at the C-terminus, and leads to binding of anti-ΔP (Figure S5). Considering this strict and limited specificity of anti-ΔP antibody, the results from the immunochemical analysis indicate that the carboxytermini of native GluA4 (or GluA2_L_) receptors do not end with DL, as predicted by the hypothesized removal of the “blocking” residues by endogenous enzyme activities.

The results of the present study refute the hypothesis of a functional cryptic PDZ motif in GluA4 and GluA2_L_ receptors and have implications for the functional relationships of long-tailed AMPA receptor subunits, and, evidently, for the interpretation of the observed association of GluA4 with SAP97. Despite their sequence-level similarities to GluA1 and related roles in synaptic plasticity, GluA4 and GluA2_L_ receptors do not engage in direct PDZ interactions. A distinct role for “non-GluA1” long-tailed subunits is supported by several recent studies which have highlighted regulatory differences between GluA1 and GluA2_L_/GluA4 in signaling pathways involving Ras [37], [38] and Jun N-terminal kinase [39]. The functional implications of coimmunoprecipitation of native GluA4 AMPA receptors with SAP97, are not clear, especially as the role of SAP97 even in GluA1-dependent synaptic plasticity is still poorly understood [11], [12], [16], [20]. As regards the mechanism of GluA4-SAP97 association, both direct non-PDZ interaction and an indirect association mediated by other proteins should be considered. Our finding that wild-type GluA4 or its C-terminal domain do not show *in vitro* binding to SAP97 ([13], and this study) would argue against a direct binary interaction; however, the situation may be different under physiological conditions. An indirect assocation would be most easily explained by common interaction partners. Stargazin and related transmembrane AMPA receptor regulatory proteins (TARP) interact with all AMPA receptor subunits and carry a type I PDZ motifs in their C-termini, giving a potential to bind to SAP97 and other Maguks [40]. It has been reported, however, that, in contrast to PSD-95, SAP97 does not coimmunoprecipitate with TARP from native tissue [41]. Another candidate interaction partner for both GluA4 and SAP97 is 4.1N, a scaffolding protein linked to cell surface transport and anchorage of GluA1 [42], [43] and GluA4 [25] AMPA receptors. In support of this possibility, we have found that 4.1N and SAP97 coimmunoprecipitate from mouse brain (Figure S6). This finding may not be unexpected as SAP97 has been shown to bind to 4.1R, another member of 4.1 family scaffolding proteins, and this binding is mediated by FERM domain, highly conserved in the 4.1 family [44], [45]. Whether 4.1N, GluA4 and SAP97 are all present in the same molecular complex, thereby explaining the coimmunoprecipitation of GluA4 with SAP97, is presently unclear.

### Conclusion

We conclude that the available evidence does not give any support for the postulated cleavage of the single carboxy-terminal blocking residue from GluA4 or GluA2_L_ subunits. If such processing takes place at all, the processed subunits would represent a negligible fraction of the total subunit pool, a notion inconsistent with a significant role in receptor trafficking or synaptic anchoring. We also found that native GluA4 AMPA receptors are associated *in vivo* with SAP97. Whether this novel interaction is direct, mediated by non-PDZ mechanisms, or indirect, mediated by other scaffolding proteins like 4.1N, TARPs and other Maguks [25], [37], [46] remains to be established.

## Materials and Methods

### Ethics Statement

Immunization (primary immunization with 300 µg protein; two boosters, 200 µg each) and collection of sera was performed according to standard protocols in the Animal Facility of the Viikki Biocenter, University of Helsinki.

### Antibodies and Peptides

Polyclonal rabbit antiserum (Anti-BD_L_) raised against a glutathione S-transferase (GST) fusion protein of GluA4 C-terminal residues 836–901, and recognizing wild-type GluA4 and GluA2_L_, has been described [35]. An antibody fraction specific for the processed C-terminus (ΔP) was isolated from anti-BD_L_ as follows. Initially, IgGs recognizing the native GluA4 C-terminal domain were removed from anti-BD_L_ antiserum through extensive incubation against GST-GluA4 CTD with native C-terminus (residues 836–902) immobilized on nitrocellulose. After removal of all immunoreactivity against wild-type GluA4 as monitored by immunoblotting, the depleted antiserum was incubated with similarly immobilized GST fusion protein containing GluA4 CTD lacking the carboxy-terminal residue proline-902 (residues 836–901). The bound anti-ΔP IgGs were eluted in 0.2 M glycine, pH 2.4, and neutralized by addition of 1 M Tris-HCl, pH 8.0. Thereafter, the antibody fractions were concentrated and buffer exchanged into PBS by using 50K Microsep Centrifugal devices (PALL Life Sciences, Ann Arbour, MI). The purified antibody was used at 0.35 µg/ml for immunoblotting and for immunofluoresence labeling at 7 µg/ml.

A generic antiserum against PSD-95 family proteins (anti-PDZ) was generated by immunizing New Zealand White rabbit with purified GST fusion protein of rat PSD-95 PDZ domains 1–3 (residues 213–549; SwissProt P31016). Immunization (primary immunization with 300 µg protein; two boosters, 200 µg each) and collection of sera was performed according to standard protocols in the Animal Facility of the Viikki Biocenter, University of Helsinki. The antiserum was used at 1∶4000 for immunoblotting.

Fab7, a monoclonal conformation-specific recombinant Fab antibody recognizing an N-terminal epitope in GluA4 and GluA2, has been described [36]. All other antibodies have been previously described, and were used at the following dilutions for immunoblotting: anti-GluR-A_CTD_ (GluA1_CTD_) (serum, 1∶2000; [46]); anti-BD_L_ (GluA2_L_/GluA4) (serum, 1∶1000; purified IgG 0.4 µg/ml; [35]); anti-D_X_ (GluA4) (serum, 1∶2000; [25]); anti-SAP97_N_ (serum, 1∶2000; [13]); anti-4.1N (serum, 1∶2000; [47]). Commerical antibodies were used at the dilutions recommended by the manufacturers: anti-flag M2 (mouse mAb; Sigma), anti-GluR4 (rabbit polyclonal IgG; Sigma), anti-GFP (mouse mAb; Sigma), anti-myc (rabbit polyclonal IgG; AbCam), anti-Dynamin (mouse mAb, AbCam). The following secondary antibodies were used; anti-mouse-HRP (1∶3000; GE Healthcare), anti-rabbit-HRP (1∶8000; GE Healthcare), anti-mouse-Cy3 (1∶100; Jackson Immunochemicals) and anti-rabbit-Rhodamine RedX (1∶100; Jackson Immunochemicals).

Synthetic GluA4 carboxy-terminal peptides (13mer, RQSSGLAVIASDL; 14mer, RQSSGLAVIASDLP; both with >95% purity) were purchased from Sigma Genosys, Cambridge, UK.

### DNA Constructs

Expression plasmids encoding N-terminally Flag- or GFP-tagged rat AMPA receptor subunits were constructed in pcDNA3.1 (Stratagene) as described earlier [25]. C-terminal mutations were introduced in the expression plasmids by PCR methodology. The regions encoding the short CTD of GluA4 (GluA4_S_) and the long CTD of GluA2 (GluA2_L_) were generated by PCR, using appropriately designed oligonucleotide primers, and subcloned into the corresponding pcDNA3.1 expression vectors. GluA4_S_ cDNA in pBluescript, kindly donated by Dr Andres Buonanno (NICHHD-NIH, Bethesda, MD), and mouse brain cDNA served as templates for the PCR reactions. A double-stranded synthetic oligonucleotide encoding the C-terminal residues 845–864 of rat dynamin-1 (Swiss-Prot P21575) was ligated between the BglII and HindIII sites of pEGFP-C1 (Clontech) to generate GFP-Dynamin1-c expression construct. All new plasmid constructs were verified by restriction mapping and by complete sequencing of PCR -amplified regions. N-terminally myc-tagged SAP97, SAP102 and PSD-95 constructs have been described [46]. Expression constructs for mLin-2 and mLin10 (in pRK5 plasmid) were generously donated by Dr. Ben Margolis (University of Michigan, Ann Arbor, MI).

### GST Fusion Protein Production and Purification

GST fusion protein constructs were generated in pGEX4-T3 vector and expressed in *E.coli* BL21 according to manufacturer's instructions (GE Healthcare). Briefly, bacterial cultures were grown at 37°C until OD_600_ of 0.6, and the protein production was induced with 1 mM IPTG for 3 hr at 30°C. Bacteria were pelleted, frozen and resuspended in PBS buffer containing 1 mM PMSF and sonicated. After centrifugation, the supernatant was incubated with glutathione-sepharose (GE Healthcare) for two hours at 4°C. After extensive washing the proteins were eluted in 10 mM glutathione in 50 mM Tris-HCl, pH 8.

### Cell Culture, Transfection and Immunocytochemistry

HEK293 and Cos-7 cells were cultured and transfected as previously described [25]. For co-expression cDNAs were transfected at a 1∶1 ratio. For immunofluoresence staining cells were grown on poly-d-lysine coated coverslips. The cells were fixed by 3% paraformaldehyde and blocked against nonspecific binding by incubation in 3% goat serum containing 0.05% Triton-X 100. Images were obtained via an Olympus Provis AX70 epifluorescence microscope coupled to a Photometrics SenSys air-cooled CCD camera.

### Immunoprecipitation

Immunoprecipitations were performed essentially as described previously [13], [25]. Briefly, transfected HEK293 cells, or 12-week mouse brains, or adult rat cerabella were homogenized in 50 mM Tris-HCl pH 8.0 containing 150 mM NaCl, 5 mM EDTA, 1% Nonidet P-40, 5 mM NaF, 1 mM Na-pyrophosphate, 1 mM Na_3_VO_4_, 2 mM PMSF and 10 µg/ml aprotinin and leupeptin (TNE buffer; 1 ml per 50–100 mg tissue). Triton-X 100 was added to a final concentration of 1% and the homogenate was mixed for two hours at 4°C, followed by centrifugation at 20000 g for 20 min at 4°C for HEK cell extracts or 100,000 g for 60 min at 4°C for brain tissue extracts. The supernatants were pre-cleared by incubation with GammaBind G-Sepharose (GE Healthcare), followed by overnight incubation with the appropriate antibody, antisera or pre-immune sera (2 µl/ml extract) at 4°C. The immune complexes were harvested by incubation with GammaBind G-Sepharose (2 h, +4°C). After three 10 minute washes with TNE buffer and two five minute washes with PBS, the proteins were eluted in Laemmli's sample buffer and resolved by SDS-PAGE. Immunoblotting was performed as described earlier [13], [25].

### Mass Spectrometry

Rat cerebellar extracts immunoprecipitated with the appropriate antibodies as described above were resolved by SDS-PAGE. Proteins were visualized by Coomassie Brilliant Blue staining and bands of interest were cut out and subjected to in-gel digestion essentially as described by Shevchenko *et al.* [48]. Briefly, proteins were reduced with dithiotreitol and alkylated with iodoacetamide before digestion with sequencing grade modified trypsin (2% wt/wt; Promega) overnight at 37°C. Peptides were extracted once with 25 mM ammonium bicarbonate and twice with 5% formic acid and the extracts were pooled. Prior to MALDI-TOF mass spectrometric analysis, peptide mixture was desalted and concentrated using Millipore μ-C18 ZipTips. Peptide mass fingerprint analysis of the generated peptides was performed with an Ultraflex MALDI-TOF/TOF mass spectrometer (Bruker-Daltonics, Bremen, Germany) in the positive ion reflector mode using α-cyano-4-hydroxycinnamic acid as the matrix. MALDI-TOF spectra were externally calibrated with the standard peptide mixture from Bruker-Daltonics (Bremen, Germany). Database searches were carried out by Mascot peptide map fingerprint search program version 2.2 (http://www.matrixscience.com/) using the following parameters: enzymatic cleavage with trypsin; one potential missed cleavage; peptide mass tolerances of ±50 ppm; modifications due to carbamidomethylation of Cys (fixed) and oxidation of Met (variable) were allowed. The initial identifications were then refined by subjecting all potential AMPA subunits and splice variants to theoretical tryptic digest with the same parameters via EXPASY Peptide Mass Tool (http://au.expasy.org/tools/peptide-mass.html). The resulting predicted peptide masses were then manually compared to the experimentally generated data.

## Supporting Information

Figure S1 Conservation of GluA2_L_ C-terminal sequence in vertebrate evolution. The indicated GluA2_L_ orthologs represent diverse vertebrate lineages: mammals (Rattus norvegicus, rat, P19490), birds (Gallus gallus, chicken, Q90858), bony fishes (Danio rerio, zebra fish, Q71E58) and amphibians (Xenopus tropicalis, western clawed frog; the sequence represents a virtual translation of Genbank EST CX366243). In the alignment, the residues conforming to the class I PDZ motif (-Thr/Ser-X-Φ; Φ denoting an amino acid residue with large aliphatic side chain, X standing for any amino acid) are highlighted in yellow. Asterisks indicate identical residues, whereas strong and weak similarities (according to Gonnet Pam250 matrix [40]) are indicated by colons and dots, respectively.
(0.25 MB TIF)

Figure S2 Characterization of anti-PDZ serum. HEK293 cell extracts of myc-tagged constructs indicated above and no DNA (mock) were immunoprecipitated with anti-myc IgG and probed with antibodies indicated to the left. Anti-PDZ recognizes PSD-95 family Maguk proteins, SAP97, SAP102 and PSD95; but not non-PSD-95 family PDZ domain-containing proteins, mLin-10 or mLin-2. In rat cerebellar tissue extract (CB) the anti-PDZ serum detects multiple bands, as expected.
(0.12 MB TIF)

Figure S3 Characterization of an antibody specific for the exposed PDZ motif in GluA4ΔP. (A) Immunofluoresence labelling of PFA-fixed Cos-7 cells transfected with the indicated constructs. All the subunits are expressed as shown by anti-flag labelling. However, anti-ΔP IgG recognises only GluA4ΔP, not wildtype GluA4. (B) Specific ablation of signal by pre-incubation of antibody with peptide. Pre-incubation of anti-ΔP IgG with molar excess of 13mer peptide prevented detection of recombinant GluA4ΔP, similar treatment with 14mer peptide had no effect (left hand panels). Conversely, anti-GluR4 was only fully blocked with the 14mer peptide (right hand panels).
(0.79 MB TIF)

Figure S4 Anti-ΔP IgG recognizes rat dynamin1 C-terminus. (A) Rat cerebellar extract was immunoprecipitated by a panel of antibodies indicated on top and the samples were probed as indicated to the left. A 100-kDa dynamin band is present in the input and in anti-ΔP immunoprecipitate, but not in anti-BD_L_ or Fab7 immunoprecipitates (upper panel). Conversely, anti-ΔP and dynamin immunoprecipitates do not contain any detectable GluR4 immunoreactivity (lower panel). (B) HEK293 extracts containing GFP-dynamin 1[845-864] fusion protein or GFP only were immunoprecipitated with anti-GFP and immunoblotted with anti-ΔP IgG. Anti-ΔP reacted strongly with the 27-kDa dynamin fusion but not with GFP (upper panel). Both proteins were similarly expressed, as shown by the anti-GFP blot (lower panel; the lower bands correspond to IgG).
(0.26 MB TIF)

Figure S5 Effect of deletion of the carboxyterminal serine residue on immunoreactivity and PDZ interactions of GluA2_L_.(A) The anti-ΔP IgG recognizes the exposed PDZ motif in GluA2_L_ΔS. HEK293 cells expressing Flag-tagged GluA2_L_ and GluA2_L_ΔS proteins were immunoblotted with the antibodies indicated below the panels. The anti-BD_L_ IgG detects both proteins, whereas anti-ΔP IgG only detects GluA2_L_ΔS. (B) Preincubation of anti-ΔP IgG with molar excess of 13mer peptide prevented detection of recombinant GluA2_L_ΔS (right hand panel). (C) GluA2_L_ΔS binds to SAP97 PDZ domains. Extracts of HEK293 cells expressing Flag-tagged GluA2_L_ and GluA2_L_ΔS were incubated with SAP97[PDZ1-3] GST fusion protein. Input (upper) panel indicates similar expression of GluA2 proteins. The lower panel shows only GluA2_L_ΔS is pulled down with the PDZ domains. Both blots were probed with anti-Flag IgG.
(0.29 MB TIF)

Figure S6 4.1N interacts with SAP97 and AMPA receptors. Mouse brain extract was subjected to immunoprecipitation with the antibodies indicated on top and the samples were probed with anti-4.1N antibody [47]. 4.1N was present in the immunoprecipitates produced by antibodies specific for SAP97, PSD-95 Maguks (anti-PDZ) and GluA2/GluA4 AMPA receptor subunits (Fab7).
(0.18 MB TIF)

Table S1 Monoisotopic peptide masses observed in the mass spectrometric analysis of ∼100 kDa band in anti-BD_L_ IgG immunoprecipitate from adult rat crebellum and theoretical mases of tryptic peptides of rat AMPA receptor subunits.
(0.04 MB DOC)

Table S2 Monoisotopic peptide masses observed in the mass spectrometric analysis of ∼100 kDa band in anti-ΔP IgG immunoprecipitate from adult rat crebellum and theoretical mases of tryptic peptides of rat dynamin isoforms. C-terminal peptide is underlined.
(0.03 MB DOC)
