# Supplementary figures and images for: Analysis of the Potential Role of GluA4 Carboxyl-Terminus in PDZ Interactions
Source: PLoS One. 2010 Jan 14;5(1):e8715. doi: 10.1371/journal.pone.0008715 (PMC2806832; doi:10.1371/journal.pone.0008715)

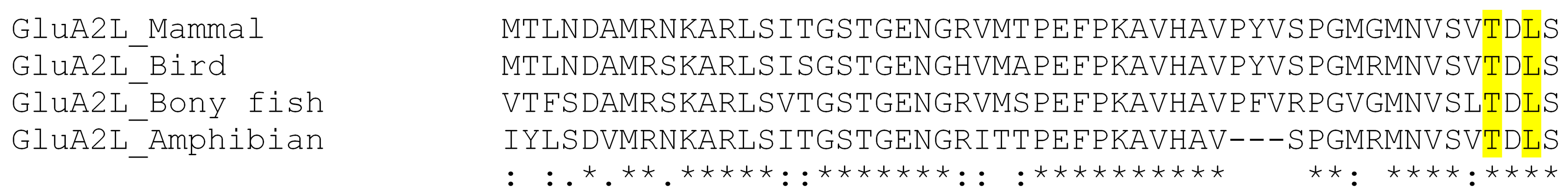

Supplement: Figure S1 — Conservation of GluA2L C-terminal sequence in vertebrate evolution. The indicated GluA2L orthologs represent diverse vertebrate lineages: mammals (Rattus norvegicus, rat, P19490), birds (Gallus gallus, chicken, Q90858), bony fishes (Danio rerio, zebra fish, Q71E58) and amphibians (Xenopus tropicalis, western clawed frog; the sequence represents a virtual translation of Genbank EST CX366243). In the alignment, the residues conforming to the class I PDZ motif (-Thr/Ser-X-Φ; Φ denoting an amino acid residue with large aliphatic side chain, X standing for any amino acid) are highlighted in yellow. Asterisks indicate identical residues, whereas strong and weak similarities (according to Gonnet Pam250 matrix [40]) are indicated by colons and dots, respectively. (0.25 MB TIF) [file pone.0008715.s001.tif]

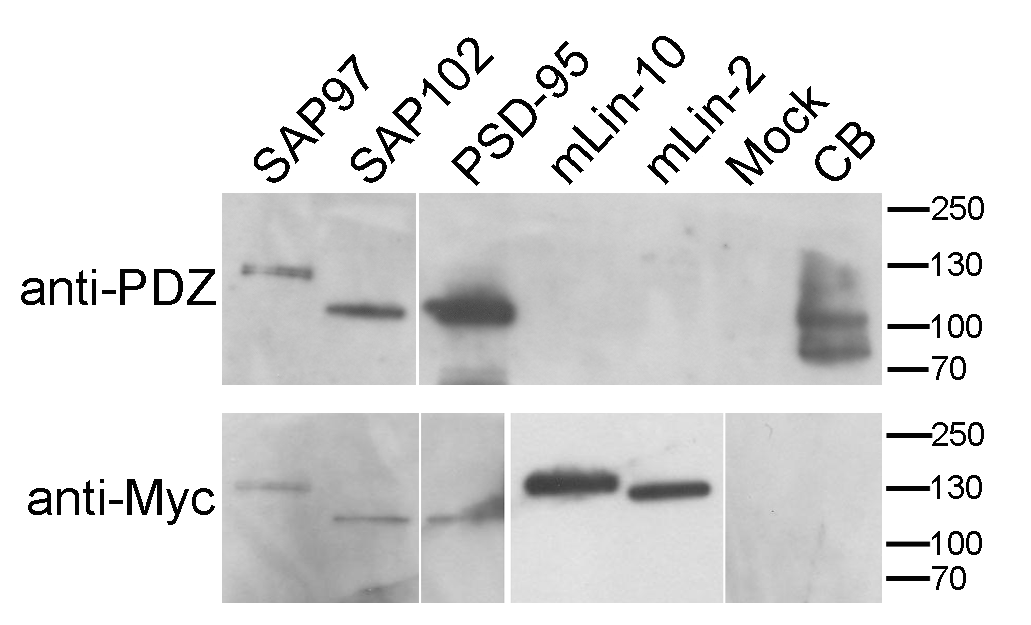

Supplement: Figure S2 — Characterization of anti-PDZ serum. HEK293 cell extracts of myc-tagged constructs indicated above and no DNA (mock) were immunoprecipitated with anti-myc IgG and probed with antibodies indicated to the left. Anti-PDZ recognizes PSD-95 family Maguk proteins, SAP97, SAP102 and PSD95; but not non-PSD-95 family PDZ domain-containing proteins, mLin-10 or mLin-2. In rat cerebellar tissue extract (CB) the anti-PDZ serum detects multiple bands, as expected. (0.12 MB TIF) [file pone.0008715.s002.tif]

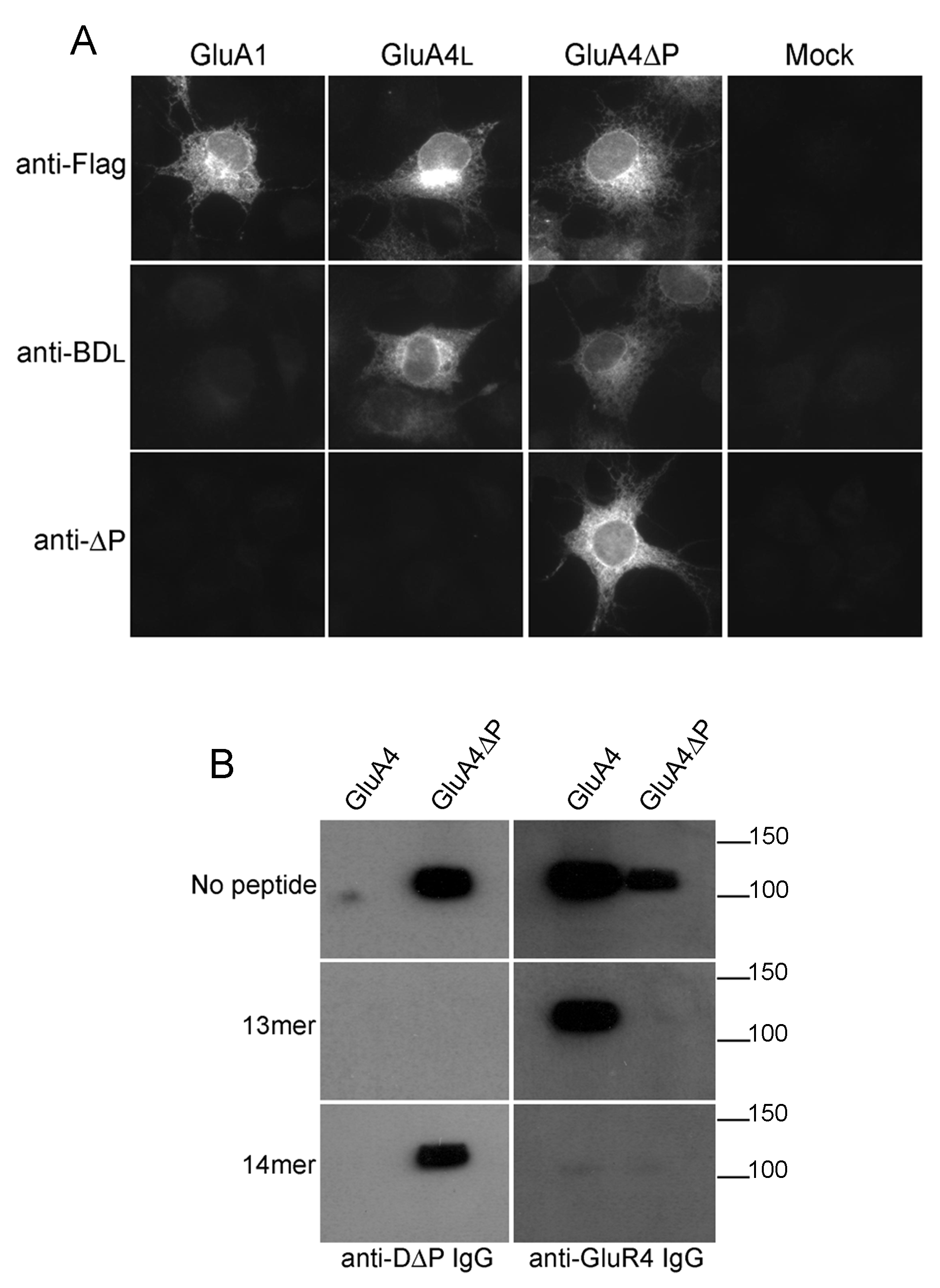

Supplement: Figure S3 — Characterization of an antibody specific for the exposed PDZ motif in GluA4ΔP. (A) Immunofluoresence labelling of PFA-fixed Cos-7 cells transfected with the indicated constructs. All the subunits are expressed as shown by anti-flag labelling. However, anti-ΔP IgG recognises only GluA4ΔP, not wildtype GluA4. (B) Specific ablation of signal by pre-incubation of antibody with peptide. Pre-incubation of anti-ΔP IgG with molar excess of 13mer peptide prevented detection of recombinant GluA4ΔP, similar treatment with 14mer peptide had no effect (left hand panels). Conversely, anti-GluR4 was only fully blocked with the 14mer peptide (right hand panels). (0.79 MB TIF) [file pone.0008715.s003.tif]

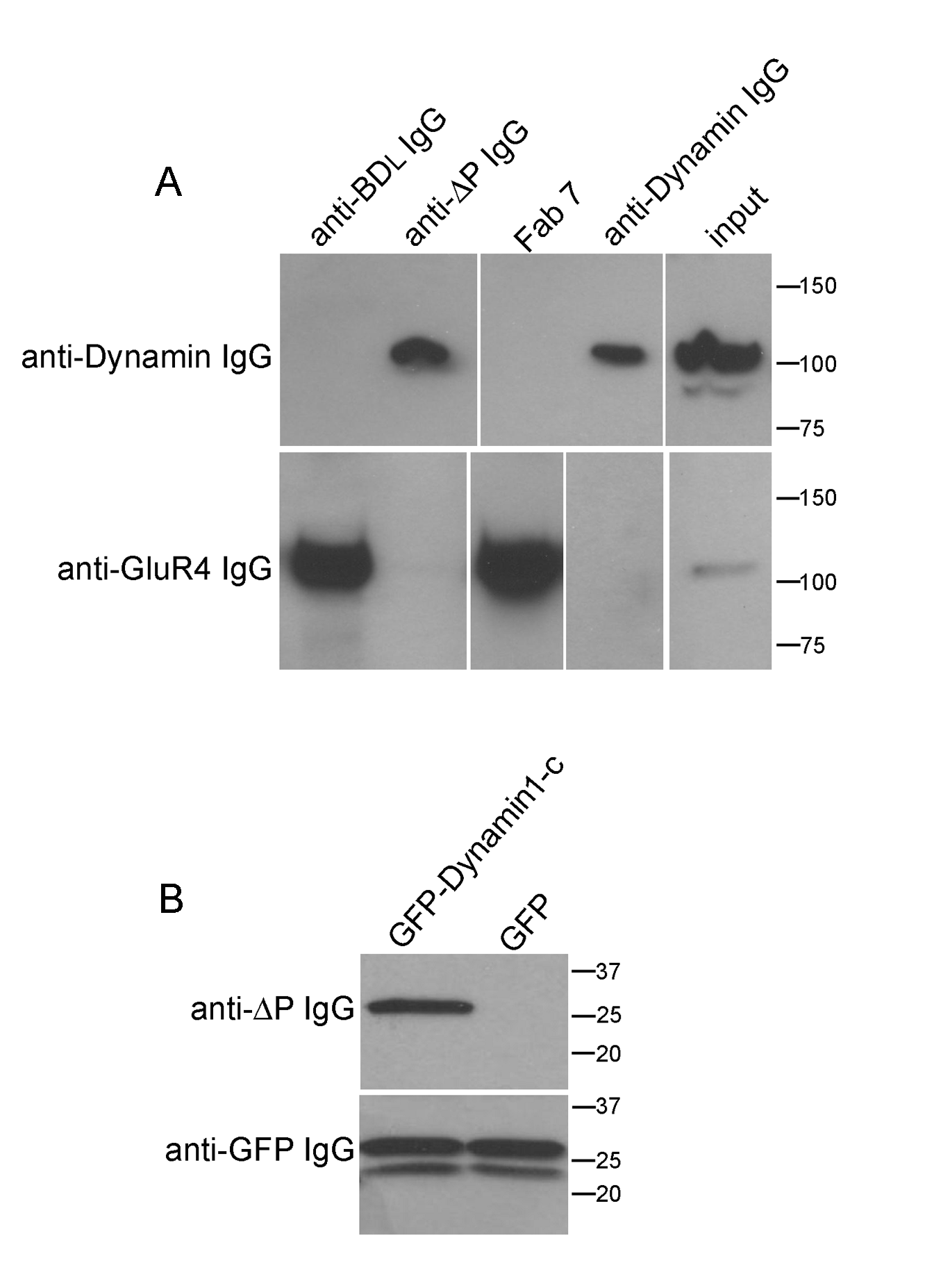

Supplement: Figure S4 — Anti-ΔP IgG recognizes rat dynamin1 C-terminus. (A) Rat cerebellar extract was immunoprecipitated by a panel of antibodies indicated on top and the samples were probed as indicated to the left. A 100-kDa dynamin band is present in the input and in anti-ΔP immunoprecipitate, but not in anti-BDL or Fab7 immunoprecipitates (upper panel). Conversely, anti-ΔP and dynamin immunoprecipitates do not contain any detectable GluR4 immunoreactivity (lower panel). (B) HEK293 extracts containing GFP-dynamin 1[845-864] fusion protein or GFP only were immunoprecipitated with anti-GFP and immunoblotted with anti-ΔP IgG. Anti-ΔP reacted strongly with the 27-kDa dynamin fusion but not with GFP (upper panel). Both proteins were similarly expressed, as shown by the anti-GFP blot (lower panel; the lower bands correspond to IgG). (0.26 MB TIF) [file pone.0008715.s004.tif]

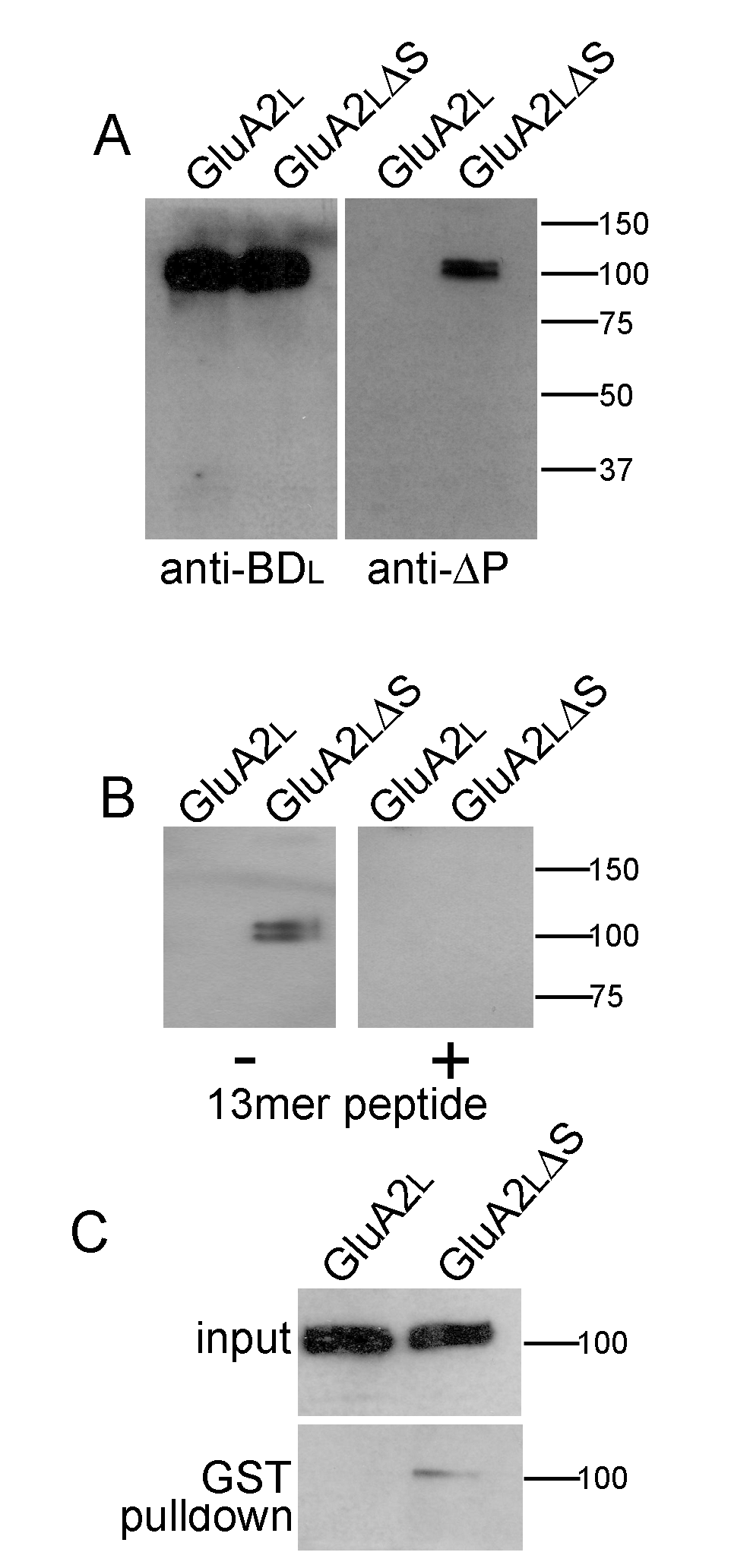

Supplement: Figure S5 — Effect of deletion of the carboxyterminal serine residue on immunoreactivity and PDZ interactions of GluA2L.(A) The anti-ΔP IgG recognizes the exposed PDZ motif in GluA2LΔS. HEK293 cells expressing Flag-tagged GluA2L and GluA2LΔS proteins were immunoblotted with the antibodies indicated below the panels. The anti-BDL IgG detects both proteins, whereas anti-ΔP IgG only detects GluA2LΔS. (B) Preincubation of anti-ΔP IgG with molar excess of 13mer peptide prevented detection of recombinant GluA2LΔS (right hand panel). (C) GluA2LΔS binds to SAP97 PDZ domains. Extracts of HEK293 cells expressing Flag-tagged GluA2L and GluA2LΔS were incubated with SAP97[PDZ1-3] GST fusion protein. Input (upper) panel indicates similar expression of GluA2 proteins. The lower panel shows only GluA2LΔS is pulled down with the PDZ domains. Both blots were probed with anti-Flag IgG. (0.29 MB TIF) [file pone.0008715.s005.tif]

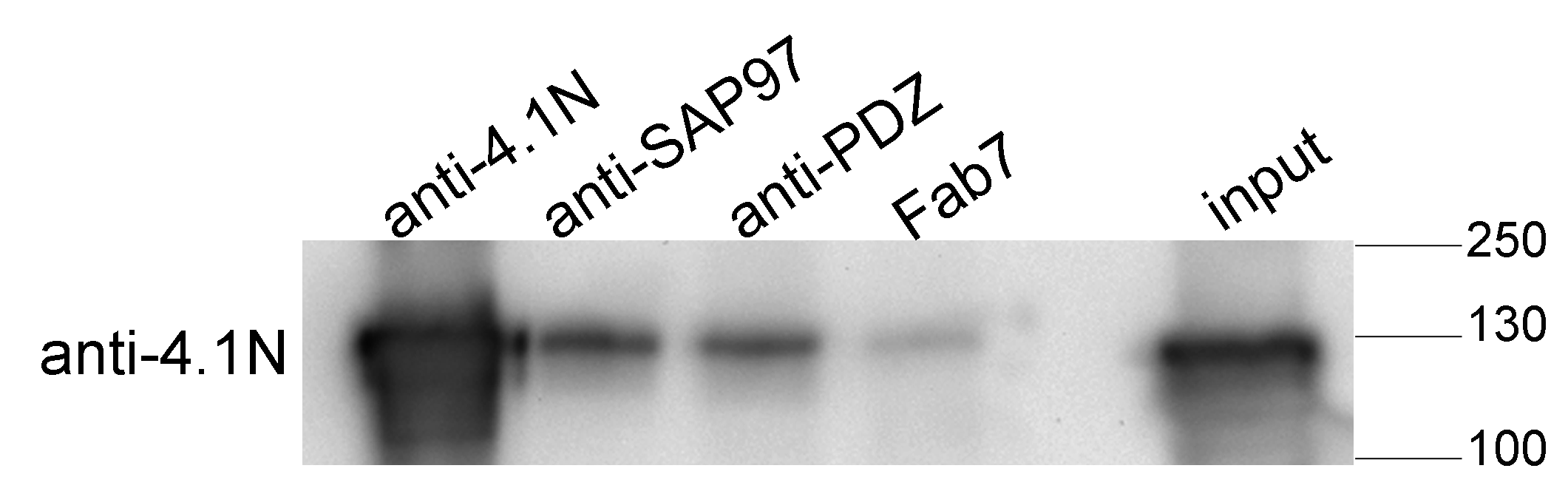

Supplement: Figure S6 — 4.1N interacts with SAP97 and AMPA receptors. Mouse brain extract was subjected to immunoprecipitation with the antibodies indicated on top and the samples were probed with anti-4.1N antibody [47]. 4.1N was present in the immunoprecipitates produced by antibodies specific for SAP97, PSD-95 Maguks (anti-PDZ) and GluA2/GluA4 AMPA receptor subunits (Fab7). (0.18 MB TIF) [file pone.0008715.s006.tif]
