# Supplementary material for: Analysis of the Potential Role of GluA4 Carboxyl-Terminus in PDZ Interactions
Source: PLoS One. 2010 Jan 14;5(1):e8715. doi: 10.1371/journal.pone.0008715 (PMC2806832; doi:10.1371/journal.pone.0008715)
Supplement: Table S1 — Monoisotopic peptide masses observed in the mass spectrometric analysis of ∼100 kDa band in anti-BDL IgG immunoprecipitate from adult rat crebellum and theoretical mases of tryptic peptides of rat AMPA receptor subunits. (0.04 MB DOC) [file pone.0008715.s007.doc]

**Table S1** Monoisotopic peptide masses observed in the mass spectrometric analysis of ~100 kDa band in anti-BD_L_ IgG immunoprecipitate from adult rat crebellum and theoretical mases of tryptic peptides of rat AMPA receptor subunits.

| Observed mass | Calculated mass | Peptide location in protein | Peptide |
| --- | --- | --- | --- |
| 2761.21 | 2761.252 | GluA1 425-448 | NANQFEGNDRYEGYCVELAAEIAK |
| 2465.15 | 2465.21  2465.213 | GluA1 317-339  GluA2 295-315 | RGNAGDCLANPAVPWGQGIDIQR  YTSALTYDAVQVMTEAFRNLR |
| 2456.06 | 2456.119 | GluA4 325-347 | RGNAGDCLANPAAPWGQGIDMER |
| 2300.01 | 2300.105  2300.018 | GluA3 856-875  GluA4 326-347 | NTQNFKPAPATNTQNYATYR  GNAGDCLANPAAPWGQGIDMER |
| 2178.09 | 2178.13 | GluA1 340-358 | ALQQVRFEGLTGNVQFNEK |
| 2022.88 | 2022.948  2022.948 | GluA1 714-729  GluA2 721-736 | YAYLLESTMNEYIEQR  YAYLLESTMNEYIEQR |
| 1745.88 | 1745.9 | GluA1 841-855 | GFCLIPQQSINEAIR |
| 1704.83 | 1704.845 | GluA4 354-368 | IQGLTGNVQFDHYGR |
| 1454.86 | 1454.869  1454.869 | GluA2 760-773  GluA4 761-774 | GSSLRTPVNLAVLK  GSSLRTPVNLAVLK |
| 1259.70 | 1259.711 | GluA4 464-475 | IAIVPDGKYGAR |
| 1195.64 | 1196.562  1195.65 | GluA3 211-219  GluA4 847-856 | YLIDCEVER  MKLTFSEAIR |
| 936.49 | 936.446  936.515 | GluA1 699-706  GluA4 849-856 | TTEEGMIR  LTFSEAIR |
