# Supplementary material for: Analysis of the Potential Role of GluA4 Carboxyl-Terminus in PDZ Interactions
Source: PLoS One. 2010 Jan 14;5(1):e8715. doi: 10.1371/journal.pone.0008715 (PMC2806832; doi:10.1371/journal.pone.0008715)
Supplement: Table S2 — Monoisotopic peptide masses observed in the mass spectrometric analysis of ∼100 kDa band in anti-ΔP IgG immunoprecipitate from adult rat crebellum and theoretical mases of tryptic peptides of rat dynamin isoforms. C-terminal peptide is underlined. (0.03 MB DOC) [file pone.0008715.s008.doc]

**Table S2** Monoisotopic peptide masses observed in the mass spectrometric analysis of ~100 kDa band in anti-ΔP IgG immunoprecipitate from adult rat crebellum and theoretical mases of tryptic peptides of rat dynamin isoforms. C-terminal peptide is underlined.

| Observed mass | Calculated  mass | Peptide location in protein  (specific isoforms) | Peptide |
| --- | --- | --- | --- |
| 2023.856 | 2023.968 | Dyn1 343-361 | RIEGSGDQIDTYELSGGAR |
| 1924.846 | 1924.951 | Dyn 1 847-864 (in 1, 3, 5, 7) | SGQASPSRPESPRPPFDL |
| 1375.646 | 1375.712 | Dyn1 584-594 | HIFALFNTEQR |
| 1335.682 | 1335.749 | Dyn 1 280-290 | VLNQQLTNHIR |
| 1256.604 | 1256.667 | Dyn 1 5-15 | GMEDLIPLVNR |
| 1240.600 | 1240.664 | Dyn 1 189-199 | IAKEVDPQGQR |
| 1175.603 | 1175.664 | Dyn 1 785-796 (in 1, 2, 4, 5, 6, 8) | APAVPPARPGSR |
| 1107.521 | 1107.579 | Dyn 1 45-54 | SSVLENFVGR |
| 1065.584 | 1065.642 | Dyn 1 114-123 | GISPVPINLR |
| 962.414 | 962.472 | Dyn 1 452-458 | LREEMER |
